# Validation of "Signs of Inflammation in Children that Kill" (SICK) score for immediate non-invasive assessment of severity of illness

**DOI:** 10.1186/1824-7288-36-35

**Published:** 2010-04-26

**Authors:** Manoj A Gupta, Anjan Chakrabarty, Ruth Halstead, Mohit Sahni, Jayanti Rangasami, Ashish Puliyel, Vishnubhatla Sreenivas, David A Green, Jacob M Puliyel

**Affiliations:** 1 Department of Pediatrics, St Stephens Hospital, Delhi, India; 2 Department of Pediatrics, West Middlesex University Hospital, UK; 3 GonzoBuzz LLP, 14 Robinson Road, 13-00 Far East Finance Building, Singapore; 4 Department of Biostatistics, All India Institute of Medical Science, New Delhi, India

## Abstract

**Objective:**

To validate the SICK scoring system's ability to differentiate between individuals with higher and lower probabilities of death

**Method:**

We performed a one year two-centre prospective evaluation of all children aged between one month and 12 years referred to the Paediatric team at St Stephens Hospital in Delhi and admitted to the Paediatric Department at West Middlesex University Hospital in London. We calculated SICK scores at presentation and correlated them with subsequent in-hospital mortality. We used discrimination by areas under receiver operating characteristic (ROC) curves to measure performance.

**Results:**

We prospectively evaluated 3895 children in Delhi and 1473 children in London. The areas under the ROC curves were 84.8% in Delhi, 81.0% in London and 84.1% (95% CI 77.4 - 90.8%) for combined data. Hosmer-Lemeshow goodness of fit for the combined data was good (Hosmer-Lemeshow Chi-square = 2.13 (p = 0.345).

**Conclusions:**

We propose the SICK score as a useful triage tool at initial presentation and highlight its particular suitability for resource poor settings.

## Introduction

The Early Warning Score [1] and Modified Early Warning Score [2] use physiological parameters to identify high risk patients on adult general wards. However they are not weighted and are based on the number of abnormal parameters. Disease specific early warning scores (e.g. CURB65 for pneumonia) have also been used in adults [3] but less so in children. The first physiological scoring system for children was the Physiology Stability Index (PSI), which derived a subjective score from the worst of 34 values from routinely measured variables over the first day on the Paediatric Intensive Care Unit (PICU) [4]. The PRISM score [5] evolved from it as a simplification with only 14 variables but still used laboratory results and so was cost and labor intensive and not assessable on presentation. The problem of lead-time bias was circumvented by the Pediatric Index of Mortality (PIM, then PIM2), which is calculated directly on admission to PICU [6]. The World Health Organization has developed emergency triage, assessment and treatment (ETAT) guidelines for use in developing countries [7] but a drawback is that these require a specific training program before implementation.

Recently Thompson and colleagues have demonstrated that vital signs can identify sick children in pediatric emergency care with comparable sensitivity to more complex triage systems [8]. They however did not develop a scoring system for use in triage. The "Signs of Inflammation in Children that Kill" (SICK) score was developed in a search for a practical triage tool for resource poor settings. Here we validate it in the context of emergency triage. The score, which evaluates the expected risk of mortality, utilizes the abnormal physical variables of the Systemic Inflammatory Response Syndrome [9] and its continuum - the Multiple Organ Dysfunction Syndrome. Its seven parameters are heart rate, respiratory rate, systolic blood pressure, temperature, blood oxygen saturation (SpO_2_), capillary refill time (CRT), and conscious level. It also takes age into account. A development study [10] of 1099 consecutive children admitted to the Paediatric Department at St Stephens Hospital (SSH) in Delhi (comprising a ward and PICU) gave regression coefficients (logs of the odds ratio of death) which are used as weightages. A previous validation study [11] at SSH was limited to 125 PICU admissions. The study we report here is a larger two-centre validation study. The objective was to validate the SICK scoring system's ability to differentiate between individuals with higher and lower probabilities of death.

## Methods

This was a prospective study from 15 November 2005 to 15 November 2006 at SSH and 6 February 2006 to 6 February 2007 at West Middlesex University Hospital (WMUH).

WMUH is a Secondary Care teaching hospital associated with the Imperial College School of Medicine. It has 400 beds and serves a population of approximately 330 000. The Paediatric Department includes a 20-bedded inpatient ward and an 8-bedded day unit but does not have a PICU. SSH is a 600-bed non-governmental hospital, which functions as a tertiary care referral centre. The Paediatric Department includes a 40-bedded inpatient ward plus a 7 bed PICU with three ventilators.

We obtained ethical approval from the SSH research committee in India and the Ealing and West London Mental Health Trust Local Research Ethics Committee in UK (REC approval reference number 05/Q0410/75). Children attending the Emergency department at SSH are first seen by an ED physician, and then referred to the Paediatric team if needed. At SSH this referral was the entry point to the study, whereas at WMUH admission or transfer to neighboring PICUs was the entry point. All patients aged between one month and 12 years (i.e. from the 29^th ^day to the 13^th ^birthday) were consecutively enrolled into the study. This represented a sample of one year's activity. At SSH we excluded patients from the analysis who left against medical advice or were referred to other hospitals (making outcome uncertain).

We evaluated SICK score parameters at entry. We measured axillary temperatures by digital electric thermometry at SSH (Dr. Morepen Digiclassic, Switzerland) and tympanic auricular temperatures at WMUH ("Genius First Time"-Infra red tympanic thermometer). We measured CRT on the sternum or a digit at the level of heart after applying blanching pressure for 5 seconds. We measured conscious level using the AVPU score ("Alert", "Responding to Voice", "Responding to Pain only" or "Unresponsive"). We measured SpO_2 _with a saturation monitor applied to the skin, usually on a finger (Larsen and Toubro Medical Stellar India at SSH; Phillips Intelli Vue MP 70 or Ohmeda Biox 3740 Pulse Oximeter at WMUH). At SSH we measured blood pressure using a sphygmomanometer cuff covering over 75% of the length of the upper arm (Larsen and Toubro Medical Stellar India) in all cases. At WMUH we measured blood pressure using an electronic blood pressure monitor (Dinamap - Critikon or Vital Signs Monitor 8100) in selective cases with the combination of increased CRT and decreased conscious level. In patients who were well with normal conscious level and CRT we did not measure BP for pragmatic reasons, on the assumption that hypotension is a late and preterminal sign in paediatric shock [12]. Doctors recorded CRT measurements and AVPU scores. Nurses measured all other parameters. The parameters used were part of the routine examination of children brought to the hospital for evaluation and so consent was not obtained for using the data. No special training of staff was required.

Abnormal ranges were taken as: heart rate > 160/minute (infant), > 150/minute (child); respiratory rate > 60/minute (infant), > 50/minute (child); systolic blood pressure < 65 (infant), < 75 (child); temperature (>38°C, <36°C); SpO_2 _(<90%); CRT (≥ 3 seconds); and conscious level (all states of consciousness except "Alert"). The variables were treated as binomial variables and classified as normal/abnormal.

The weightages taken from the development study [11] were: heart rate (0.2); respiratory rate (0.4); systolic blood pressure (1.2); temperature (1.2); SpO_2 _(1.4); CRT (1.2); conscious level (2.0); age bands <1 year (1.0), 1-5 years (0.3) and >5 years (0.0). The range of possible scores was therefore 0 to 8.6.

We calculated SICK scores by adding the weightage of each abnormal variable using custom-made software 'SICK score calculator' http://jacob.puliyel.com/sick.php. The software defaulted unmeasured parameters as normal. SICK score was not used to make clinical decisions. We followed all patients to discharge. We measured outcome as survival to discharge home or death.

Logistic regression was applied to the outcome using the calculated SICK score as the predictor. We tested the predictive ability of the SICK score by looking at the area under the receiver operating characteristic (ROC) curve. The fitted model was also assessed for its goodness of fit by applying the Hosmer-Lemeshow chi-square test, based on equally sized groups (analogous to C statistic).

## Results

At SSH, 4116 patients qualified for inclusion. Of these 108 left against medical advice and 113 transferred to another hospital. Thus, we included 3895 children in the analysis. Fifty eight children died.

At WMUH, 1473 patients were admitted during the study period and we excluded none. Five children died.

The age distributions were similar in the two centers. (Age bands <1, 1-2, 2-5, 5-10 and 10-12 years accounted for 35% versus 31%, 16% versus 20%, 23% versus 27%, 19% versus 18% and 6% versus 3% in Delhi and London respectively).

Tables 1 and 2 show observed versus expected mortalities at ascending levels of SICK score in Delhi and London respectively. Distribution of SICK scores between the two populations is significantly different (p < 0.001). Table 3 shows the fitted model for the combined data to assess for goodness of fit by applying the Hosmer-Lemeshow chi-square test. The differences between the observed and expected number of deaths and survivals were statistically not significant in the 4 risk strata. Hosmer-Lemeshow Chi-Square with 2 degrees of freedom was 2.13 (p = 0.345).

**Table 1:** Observed versus expected mortalities at ascending levels of SICK score (Delhi)

SICK Score	Delhi (SSH)			
	
	Died		Survived	
	
	Observed	Expected	Observed	Expected
0	4	1.9	805	807.0

< 1	2	3.5	1031	1029.5

1 - 1.9	8	13.0	1567	1562.0

2 - 2.9	13	8.2	334	338.8

3 - 3.9	3	3.8	60	59.2

4 - 4.9	6	3.7	19	21.3

≥ 5	22	23.8	21	19.2

**Total**	**58**	**57.9**	**3837**	**3837.0**

**Table 2:** Observed versus expected mortalities at ascending levels of SICK score (London)

SICK Score	London (WMUH)			
	
	Died		Survived	
	
	Observed	Expected	Observed	Expected
0	1	0.0	246	247.0

< 1	0	0.1	394	393.9

1 - 1.9	0	0.6	573	572.4

2 - 2.9	0	0.8	193	192.2

3 - 3.9	1	0.9	47	47.1

4 - 4.9	2	1.4	13	13.6

≥ 5	1	1.1	2	1.9

**Total**	**5**	**4.9**	**1468**	**1468**

**Table 3:** The fitted model for the combined data looking for 'goodness of fit' applying the Hosmer-Lemeshow chi-square test

		Died		Alive			Total
Group	Probability	Observed	Expected	Observed	Expected		Chi-square* (p value)
1	0.0023	6	4.4	2238	2239.6	2244	2.13 (0.345)
2	0.0047	6	5.3	1223	1223.7	1229	
3	0.0078	3	6.0	885	882.0	888	
4	0.8863	48	47.2	959	959.8	1007	

*Hosmer-Lemeshow Chi-Square with 2 degrees of freedom

**Figure 1:**
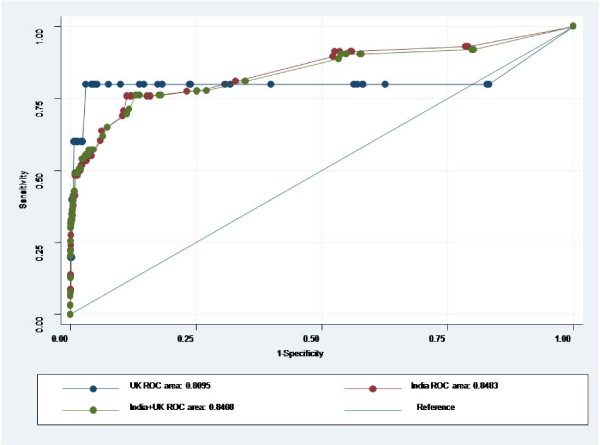
**Areas under ROC curves**.

**Figure 2:**
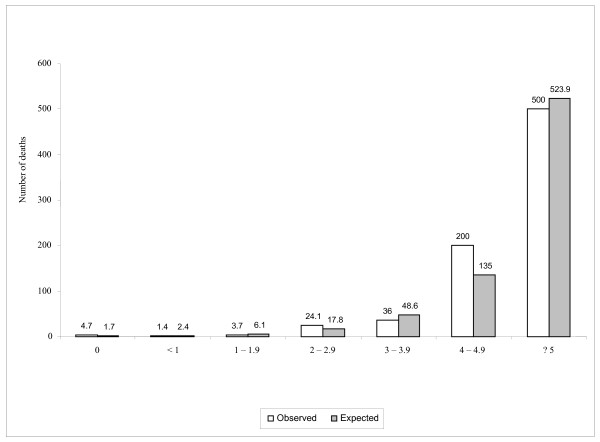
**Observed and Expected number of deaths per 1000 in each score category**.

The scoring system performed equally well in both centers. The areas under the ROC were 84.8% (95% CI: 78.2% - 91.5%) in Delhi, 81.0% (95% CI: 45.4% - 100.0%) in London and 84.1% (95% Confidence Intervals 77.4 - 90.8%) for the combined data set (figure 1). Figure 2 shows the expected and observed mortality (per 1000 in each score category) increasing with SICK score.

## Discussion

An important advantage for clinical practice is that the SICK score can be generated immediately on presentation. Mortality was higher in India than in the UK for any given SICK score (except 6 - 6.9, which was represented by a single patient in the UK). This would be expected and explained by differences in individual health conditions (e.g. nutritional status) and locally available resources.

The calibration of a model evaluates the degree of correspondence between the estimated probabilities of mortality it produces and the actual mortality experience of the patients. It can be statistically evaluated using "goodness-of-fit" tests [13]. Despite the difference in mortality, the calibration of the SICK score (comparing observed and expected mortality at different grades of severity) at the two centers was good. Moreover, our objective was to differentiate between individuals with higher or lower probabilities of death, for which the desired test quality is a good discriminatory capacity (area under ROC curve). The area under ROC curve of 84.1% for combined data confirmed a good correlation of outcome with SICK score. Furthermore, the areas under ROC curves were consistent with those seen in the development cohort (89%) [9] and the first validation study (76%) [10]. These findings imply a high probability of external validity and generalisability. Its performance in identifying sicker patients lends it to use in emergency triage.

Severity-of-illness scores have been developed for the purposes of comparative audit (comparing actual with expected outcomes over different units), evaluative research (adjusting for differences in case mix or as an aid to stratification in randomised control trials) and clinical management of individual patients (as a triage tool or as a clinical shorthand to rapidly convey patient information). However, the latter purpose has been addressed by very few paediatric scoring systems. An ideal triage scoring system must be available immediately on presentation and the score must accurately describe the severity of illness. The overall goals of triage are to determine if a patient is appropriate for a given level of care and to ensure that hospital resources are utilized effectively.

We measured temperatures in the axilla at SSH and tympanically at WMUH because this was the standard practice in the two units at the time. Studies have shown that axillary temperature is lower than core temperature: a systematic review of studies found a pooled mean temperature difference of 0.85°C for digital electric thermometers [14]. This difference would tend to skew the data towards a relative under diagnosis of fever and over diagnosis of hypothermia at SSH compared to WMUH (although the SICK score would be the same for each category). We felt that for practical purposes this did not make an important difference to score distributions.

We used normal values as default readings for missing values. Consequent misclassifications would spuriously lower the observed sensitivity of the score and would therefore not detract from the satisfactory performance shown by the areas under the ROC curves.

The small number of deaths in the UK arm (only 5) explains the shape of its curve. Out of these 5 deaths, one child had a score of zero and the remaining 4 had scores of ≥ 3. In between these scores (> 0 and < 3) lay most (nearly 80%) of the cohort, where the absence of deaths effectively locked the sensitivity and flattened the curve. A smoother curve would have been expected with a larger number of events (in this context, deaths), which would have been more likely to be spread over different SICK scores.

We have not proposed cutoff scores to categorize patients. In order to do this a database with a large number of events (deaths) would be needed. Where most children presenting to hospital do not die, this would necessitate a huge sample size, certainly much larger than the combined numbers in this study. Furthermore, although cut off scores are necessary for the practical application of a triage system, these will vary in accordance with local conditions. These in turn reflect the balance between risk aversion and the costs and drawbacks of hospital admission in the context of available resources. We tested various cut-off values. The best cutoff for this total data was 1.5 with 77.8% sensitivity & 74.8% specificity. If the cut-off is set at 2 the sensitivity is 76.2% and specificity is 87.0% and at 2.5 the sensitivity is 57.1% and the specificity is 95.5%.

## Conclusion

The SICK score uses only physical criteria without needing recourse to laboratory results. This has cost implications and also makes it immediately determinable on presentation. Furthermore, no special training is needed for its implementation. On this basis we propose it for consideration as a triage tool in resource poor settings.

## Competing interests

The authors declare that they have no competing interests.

## Authors' contributions

MAG, JR, DAG and JP planned this two-centre study. MAG and MS were responsible for the data collection and analysis in Delhi and AC and RH for that in London. VS was the statistician for the project and AP developed the computer software - the 'SICK Score Calculator'. MAG, DAG and JP were responsible for the initial draft which was revised with input from the authors. All authors read and approved the final manuscript.
